# The Effectiveness of FES-Evoked EMG Potentials to Assess Muscle Force and Fatigue in Individuals with Spinal Cord Injury

**DOI:** 10.3390/s140712598

**Published:** 2014-07-14

**Authors:** Morufu Olusola Ibitoye, Eduardo H. Estigoni, Nur Azah Hamzaid, Ahmad Khairi Abdul Wahab, Glen M. Davis

**Affiliations:** 1 Department of Biomedical Engineering, Faculty of Engineering, University of Malaya, Kuala Lumpur 50603, Malaysia; E-Mails: marufibitoye@yahoo.com (M.O.I.); khairi@um.edu.my (A.K.A.W.); 2 Department of Biomedical Engineering, Faculty of Engineering and Technology, University of Ilorin, Ilorin, P.M.B. 1515, Nigeria; 3 Clinical Exercise and Rehabilitation Unit, The University of Sydney, Sydney, 2006 NSW, Australia; E-Mails: ehestigoni@yahoo.com.br (E.H.E.); glen.davis@sydney.edu.au (G.M.D.)

**Keywords:** evoked electromyographic (eEMG) signal, FES-evoked contraction, muscle force, muscle fatigue, M-wave, Spinal Cord Injury (SCI)

## Abstract

The evoked electromyographic signal (eEMG) potential is the standard index used to monitor both electrical changes within the motor unit during muscular activity and the electrical patterns during evoked contraction. However, technical and physiological limitations often preclude the acquisition and analysis of the signal especially during functional electrical stimulation (FES)-evoked contractions. Hence, an accurate quantification of the relationship between the eEMG potential and FES-evoked muscle response remains elusive and continues to attract the attention of researchers due to its potential application in the fields of biomechanics, muscle physiology, and rehabilitation science. We conducted a systematic review to examine the effectiveness of eEMG potentials to assess muscle force and fatigue, particularly as a biofeedback descriptor of FES-evoked contractions in individuals with spinal cord injury. At the outset, 2867 citations were identified and, finally, fifty-nine trials met the inclusion criteria. Four hypotheses were proposed and evaluated to inform this review. The results showed that eEMG is effective at quantifying muscle force and fatigue during isometric contraction, but may not be effective during dynamic contractions including cycling and stepping. Positive correlation of up to *r* = 0.90 (*p* < 0.05) between the decline in the peak-to-peak amplitude of the eEMG and the decline in the force output during fatiguing isometric contractions has been reported. In the available prediction models, the performance index of the eEMG signal to estimate the generated muscle force ranged from 3.8% to 34% for 18 s to 70 s ahead of the actual muscle force generation. The strength and inherent limitations of the eEMG signal to assess muscle force and fatigue were evident from our findings with implications in clinical management of spinal cord injury (SCI) population.

## 1. Introduction

Muscles fatigue rapidly during functional electrical stimulation (FES)-evoked spinal cord injury (SCI) muscle activation. Hence, the safety of FES systems is dependent on their ability to reduce the occurrence of muscle fatigue and to assess the condition of the stimulated muscles during FES-evoked activity sessions [1]. The ability to estimate or predict muscle force externally [2] allows the application of the decline in the muscle force to assess muscle fatigue [3]. Clinically, an optimization of the force production during evoked contractions indicates an increased fatigue resistance [4] leading to an improved rehabilitation outcome. However, given the highly non-linear and time variant nature of dynamic muscle contractions evoked by FES, the quantification of such muscle behavior is complex because of neurophysiological factors [5], such as muscle fatigue. Consequently, such muscles are unable to sustain a maximum force even in the presence of a constant stimulus [6]. A validated viable solution is an adaptive predictive control of an electrical stimulation of muscle contraction where the expected muscle force is estimated in advance [7]. Thus, an understanding of the central role of muscle forces is crucial to describing muscle activity during functional activities [8] because the initiation and sustenance of functional activities primarily involve an effective generation and coordination of the muscle force [9].

Due to the lack of proprioceptive feedback that characterizes paralyzed muscle activities, it is imperative to monitor generated muscular forces during FES-evoked activity [10]. An accurate assessment of the muscle force has become necessary for an objective evaluation of the efficacy of an FES system's intervention in rehabilitation exercises [11]. The most common assessment modality is the electromyographic (EMG) signal [12], which measures the electrical manifestation of the neuromuscular activities associated with a contracting muscle. The signal is thought to be complex as it is affected by the anatomical and physiological properties of the muscle, the control scheme of the peripheral nervous system, and the characteristics of the instrumentation used to detect and observe the signal [13]. The synchronously generated EMG signal during FES-evoked muscle contractions allows the assessment of peripheral properties of the neuromuscular system. The signal is the sum of all (*i.e.*, compound) action potentials, or M-waves, obtained directly from the evoked electromyogram (eEMG) [14]. Though the signal is rich in the muscle activation information, which is essential to understanding normal muscular activities and neuromuscular abnormalities and has been shown to correlate with the force generated during FES-evoked muscle contraction [3], the information about the sources of the artifact that readily distort the information derived from the signal in FES-evoked contraction is equally evident [15].

Typically, the non-physiological recruitment of motor units (leading to a rapid fatigue effect in FES-evoked contractions of the atrophied SCI muscles) is partly responsible for the reduced efficiency of FES systems [16]. Thus, investigators have characterized the estimation of muscle fatigue from force and strength assessments in relation to the underlying eEMG parameters [15,17] during static and dynamic muscle activities [18,19]. However, the reliability of the muscle electromyographic signal to estimate FES-evoked muscle activities using decomposed EMG signals remains inconsistent [20]. A high sensitivity to electrode placement and electrical noise, and the difficulties in the interpretation of the decomposed data continue to limit the application of the signal to support clinical decisions [21] during fresh [20] and fatiguing contractions [22].

The proven clinical relevance of FES-evoked muscle activities has led to an increased number of studies investigating the optimization of such interventions in SCI population. Therefore, this systematic review was undertaken to critically appraise the effectiveness of the evoked EMG in characterizing force and fatigue during FES-elicited muscle contractions with a special focus on the studies involving subjects with SCIs. This review does not relate, except for a brief note, to more comprehensive physiological explanations of the relationship between the motor unit and the pattern of eEMG and other technical exploratory information as could be found in the previous reviews [23–25] dedicated for those purposes. This attempt investigated topical issues related to the state of the art evidence in achieving the discrimination of the fatigue phenomenon in SCI population in view to highlight the status of an automated fatigue resistance FES-evoked contraction. Recommendations generated from this review are meant to influence the decision-making in clinical practices and facilitate the application of FES technology as a rehabilitation tool in assistive technology [26] and in exercise science [27].

## 2. The Review

### 2.1. Search Strategy

At the outset, up to 2867 peer-reviewed citations were selected from IEEE Xplore, IOP Science, MEDLINE, ScienceDirect, Scopus, SpringerLink, PubMed, and Nature electronic databases and the Google Scholar™ search engine to determine the current scope of the topic. Relevant citations were obtained between 1977 and 2014 using relevant keywords including myoelectric signal, FES-evoked contraction, muscle force, muscle fatigue, quantification, estimation, assessment, and M-wave, in various combinations. The most common abbreviations and synonyms for the terms (e.g., “elicited” for “evoked”) were also included on the search terms list. Comprehensive initial search results included journal articles, conference papers and proceedings, book chapters, and reports. Only English language citations were considered.

### 2.2. Eligibility Criteria

The electronic search was made in a broad manner, aiming to not prematurely exclude potential articles. The results, therefore, incorporated a large number of non-applicable studies. All titles and abstracts were investigated in a second round of filtering. The abstracts of identified studies were screened based on either one or more of the following inclusion criteria, which followed the central focus of this review: (i) participants were engaged in FES training for either fitness benefits, clinical outcome or functional gains based on the categorization of FES benefits of Nightingale and colleagues [28]; (ii) studies conducted to either quantify muscle force/torque or assess fatigue using FES-evoked contractions in individuals with SCI. Studies were excluded if non-human subjects were recruited or unclear protocol or data were presented.

### 2.3. Extraction of Data

The first and second authors (MI and EE) extracted data based on the objectives of the study, while NH and AA verified the validity of the information before a final compilation. GD checked the technical soundness, clarity and the flow of the study. Only study outcomes on either (i) evaluations of one of the potential benefits of FES or (ii) assessments of muscle force and fatigue, with clear objectives were retained. Information inferred from each article included topic, objectives, stimulation protocol and the type of detection electrode, the methodology adopted, result presentation, study outcomes, and suggestions for future studies.

### 2.4. Validity Assessment

All authors were eventually involved in the extraction of data from the identified citations to reduce the risk of bias. Most reported studies adopted different methods of sample selection and small sample sizes. In some retrieved articles, authors targeted both the SCI population and healthy volunteers. These studies resulted in insufficient evidence to draw definitive conclusions for generalizations on SCI population. A meta-analysis was not conducted because of wide variations in the types and protocols of the FES training, trials, and subject heterogeneity.

## 3. Results

### 3.1. Included Studies

**Figure 1.**
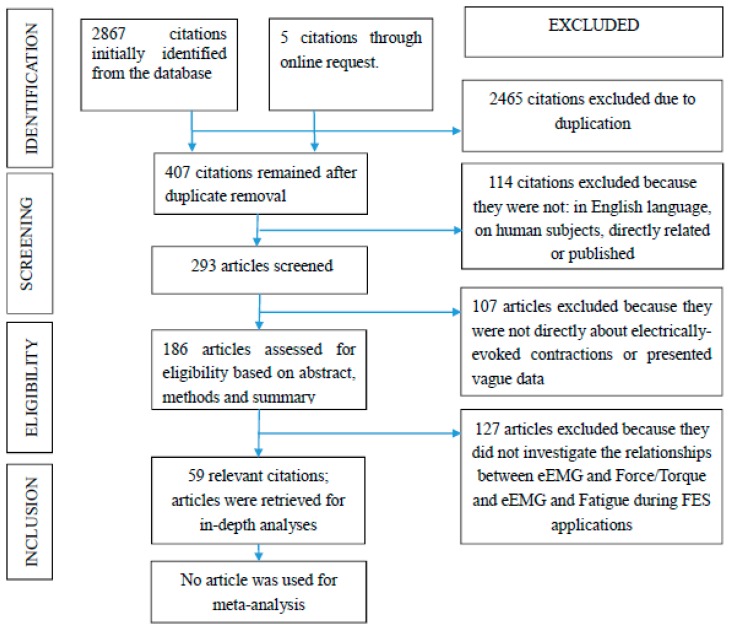
Flowchart of steps taken for the selected articles.

Out of 2867 citations retrieved, only fifty-nine satisfied the inclusion criteria (Figure 1). Twenty-two of the studies reported at least one of the following outcomes: fitness benefits, clinical outcomes assessed through improvements in the physiological well-being and functional gains assessed through the functional efficiency. The remaining thirty-five studies did not report any specific clinical, functional, or fitness outcomes of the FES-evoked muscle contraction. The main objective of this paper is addressed by the first section: (i) muscle force/torque quantification and fatigue assessments (16 studies; Tables 1 and 2). Other inert challenges that may explain intrinsic characteristics and limitations of evoked EMGs are discussed under three other subsections: (ii) evoked electromyographic signal acquisition challenges (17 studies), (iii) differences between electrically evoked and voluntary muscle contractions (six studies), and (iv) the M-wave of the eEMG during FES-evoked contraction (13 studies). The last section discusses the clinical relevance of an effective eEMG (M-wave; seven studies). The following hypotheses were tested to highlight the current state of evidence in the literature: h1: the evoked EMG signal changes with the progress of muscle fatigue during FES; h2: M-wave changes are associated with the loss of muscle force during a fatiguing FES; h3: paralyzed and healthy muscles present similar behavior in the presence of muscle fatigue during FES; and h4: M-wave parameters can be used as muscle fatigue indicators during electrically elicited contractions.

### 3.2. FES-Evoked Contraction and Muscle Force Quantification

**Figure 2.**
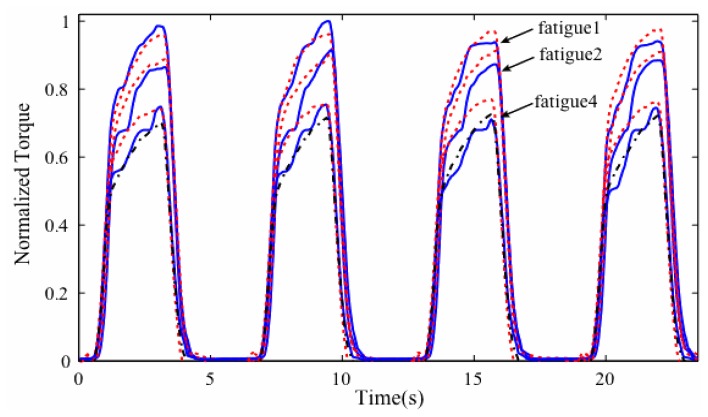
An example of a predicted torque obtained by eEMG-torque model using FES evoked fatigue induced protocol in SCI population. The blue solid line and the red dotted represent the measured and predicted torque, respectively [41].

Physiologically, a muscle contraction force originates from the global activity of the underlying muscle fibers. Evoked EMG captures information about electrical activities of motor units located at the site of the surface detection electrode. Because the electrode does not sufficiently cover the entire muscle area, the eEMG is unable to obtain the entirety of the generated signal [29] and may not serve as the optimal metric of the entire muscle force generated. The precise prediction of muscle force is thought to be a precursor to achieving efficient control of FES systems [30] because feedback information in the form of eEMG biopotential can be established between the muscle force and the muscle activation [31]. Various studies have empirically demonstrated the relationship between eEMG and muscle force/torque under both surface and implanted electrical stimulation (Table 1). To date, the lack of precise assessment of muscle force constitutes a significant knowledge gap in biomechanics due to several unresolved challenges [32], which include invasive experimental set ups, non-linearity issues, and difficulty in attaching force sensors directly on tendons or muscles [33]. Attempts to resolve these issues include analyses of inverse dynamics and dynamometry joint torque measurements [34], complete predictive simulations [35], applications of static optimization [36], applications of direct electromyogram pattern [37], analyses of electromyogram envelope extraction [38], and models driven by electromyogram [39]. The lack of consensus regarding the precise pattern of relationship between the eEMG signal and muscle output may be due to the low signal-to-noise ratio of the signal during evoked contraction, nonlinear characteristics of muscle force *versus* the velocity of shortening, the instability of muscle length under dynamic contraction, actions of agonist and antagonist muscles, and other factors depending on the neurophysiological process [40]. Consequently, limited achievable successes were evident in isometric contractions (see Figure 2) and as tabulated in Table 1, while the actual prediction or estimation of an electrically evoked muscle force during a dynamic muscle action by the underlying eEMG signal, which is vital in the efficient control of an FES assistive scheme, is currently inadequate.

With a sufficient justification for the real-time detection and the subsequent compensation for a rapid deterioration of the muscle force during an FES assisted gait [42], the current evidence suggests a limited ability of the stimulus eEMG to produce the sufficient muscle state information on the rate of muscle force deterioration during FES-evoked muscle activity [6].

### 3.3. FES-Evoked Contraction and Muscle Fatigue Assessments

Irrespective of the muscle action, muscle fatigue always precludes the optimization of the FES evoked activities in individuals with SCI. Muscle fatigue can be estimated from a decline in the maximal muscle force or the power measured instantly after fatiguing muscle contractions [47]. Logically, the automatic monitoring of fatigue during an evoked muscle activation is fundamental to the optimization of an FES system [48,49]. The force decline (and that of its derivatives) and the joint angle have been widely used to quantify fatigue, especially during the isometric contraction in paralyzed limbs [50]. Investigators have explored the parameters of the M-wave (eEMG) by directly measuring the force response of the muscle of interest during an FES-evoked fatiguing contraction to study the relationship pattern between the declining force and the underlying M-wave parameters.

The peak to peak (PTP) amplitude, root mean square (RMS) amplitude, mean frequency (MNF) and median frequency (MDF) parameters of the eEMG signal have been widely investigated to serve as muscle fatigue indices in FES-evoked contraction activities [43]. While the amplitude-based parameters have been demonstrated to be more reliable, the need to overcome the inherent stimulus artifact continues to preclude the achievement of a sufficient reliability. Only a few studies reported estimations of muscle fatigue during dynamic activities; thus, we were unable to determine the predictive accuracy during the practical application of an FES (Table 2). This implies that the conventional approach, which is based on the application of eEMG parameters as biofeedback descriptors, could not sufficiently assess muscle fatigue during functional activities, such as cycling [51,52]. The FES control of standing and stepping remains rudimentary with respect to an eEMG signal [53].

The inherent characteristics, strengths and limitations of eEMG and the theoretical explanation of the disparity observed in some experimental results in force quantification and fatigue assessments during FES-evoked contraction are discussed in the following sections.

### 3.4. Evoked Electromyographic Signal Acquisition Challenges

#### 3.4.1. Technical Challenges

Evoked EMG signals can be acquired by means of intramuscular (invasive) or surface (non-invasive) electrodes. Technically, the difference between the two modes of acquisition is due to the effect of the volume conductor, *i.e.*, the transmission of electrical information through biological tissue towards the sensor between the muscle fibers and the detection electrode. Due to the closer proximity of the electrode inserted during intramuscular detection, it is often more selective because the effect of the volume conductor is minimal [21]. The intramuscular measurement of myoelectric signals possesses richer information regarding muscle activities but is less often utilized during dynamic muscle activities because of the inherent invasiveness and difficulty in repositioning the needle electrode for repeated insertions. Thus, a limited number of active motor units can be detected due to the high selectivity of the needle electrode [20]. Neuromuscular activity measured from the surface electromyogram is limited due to the low-pass filtering induced by an anisotropic volume conductor. Hence, the bandwidth of the surface EMG signal is often reduced to less than 400 Hz (compared with 1000 Hz in intramuscular EMG) [21].

Notable research efforts on technical improvements to enhance the reliability of the information drawn from the processed electromyographic signals in muscle research have been demonstrated [57–60]. The quantitative validation of the effects of the EMG electrode placement on the muscle belly on EMG amplitude and muscle torque relationship was verified to generate a maximum value of the median frequency and the most stable muscle conduction velocity [24,61,62]. The surface EMG for the non-invasive assessment of muscle (SENIAM) procedure founded in Europe is an evidence of the concerted effort of researchers to continuously improve the efficiency of the acquired EMG signal and to ascertain the optimal electrode configuration in order to generate the highest pick-up sensitivity [63]. Investigators continued to note that the electromyographic signal is sensitive to sensor placement and is highly susceptible to noise, especially if positioned inaccurately. However, during transcutaneous (surface) FES-evoked contractions, the EMG detected by surface electrodes are often saturated by the high amplitude “noise” due to the close proximity of the electro-stimulation to the EMG detection site [64]. Therefore, the removal of such electrical stimulation artifacts, which consistently characterize and dominate acquired eEMG signals, continues to receive research attentions.

#### 3.4.2. Physiological Challenges

The information derived from a raw EMG signal is useful if it can be quantified and classified [65]. The acquisition of eEMG signals is affected by physiological parameters including motor unit recruitment, the muscle temperature, the muscle length and the cross-sectional area [66]. The issue of EMG sensitivity to cross-talk is prevalent in EMG acquisition due to activities of neighboring agonist and antagonist muscles, especially during dynamic contractions [67]. It is often difficult to identify the origin of the signal in a contraction where two or more muscles in close proximity are concurrently activated [68]. The thickness of the tissue between the muscle and electrode has also been identified as an intrinsic factor of the muscle, which has a direct effect on EMG frequency and amplitude characteristics [69]. Additionally, muscle contractions during dynamic muscle activities can potentially displace the sensor position [70], thus, making precise electrode placement practically unachievable. Hence, the acquisition of a reliable EMG signal involves the scrupulous application of an effective artifact, a noise reduction module and electrical grounding procedures [71].

### 3.5. Differences between Electrically Evoked and Voluntary Muscle Contractions

**Figure 3.**
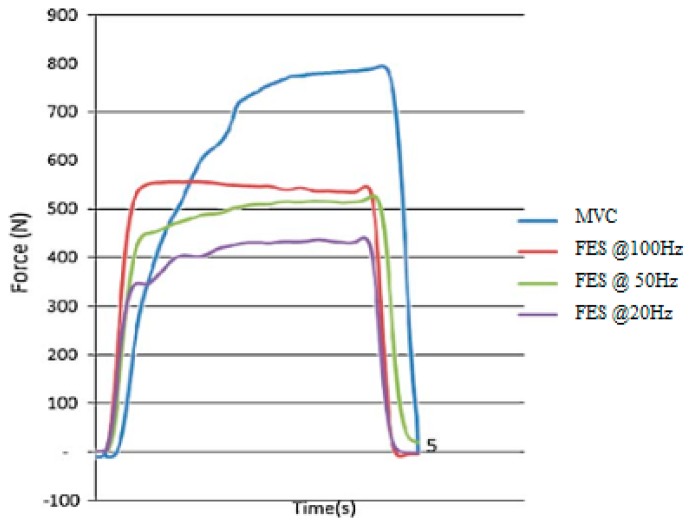
Quadriceps femoris muscle force under maximum voluntary contraction (MVC) and electrically evoked contraction (FES) at 100 Hz, 50 Hz and 20 Hz stimulation frequency [73]. The graph shows that the force generated against time in electrically evoked contraction depends on the stimulation frequency.

One of the optimal goals of the research in assistive technology for electrically evoked contraction is to achieve paraplegic muscular functions that resembles voluntary contractions [72]. However, between voluntary and electrically elicited contractions, the order of the motor unit recruitment is different [15]. At a relatively low force level, an electrical stimulation of the muscle nerves can activate fast motor units [72], unlike the typical activation pattern of motor units in voluntary contractions. Furthermore, the recruitment order of an electrically evoked contraction depends on stimulation parameters, muscle characteristics, the geometry of the stimulation electrode and certain external conditions [73], such as the frequency of stimulation (Figure 3), whereas voluntary contraction only depend on muscle intrinsic factors. The “unnatural” concurrent activation of motor units in an electrically evoked contraction leads to a general limitation of all non-physiological induced contraction, *i.e.*, a rapid development of fatigue leading to muscular inefficiency [74]. However, recent progress on optogenetics is thought to improve the pathological brain circuitry beyond nonhuman primate [75]. The unanticipated elusive goal on the orderly recruitment of motor units may have broad impact in FES assisted therapeutic and functional intervention in SCI individuals [76].

**Figure 4.**
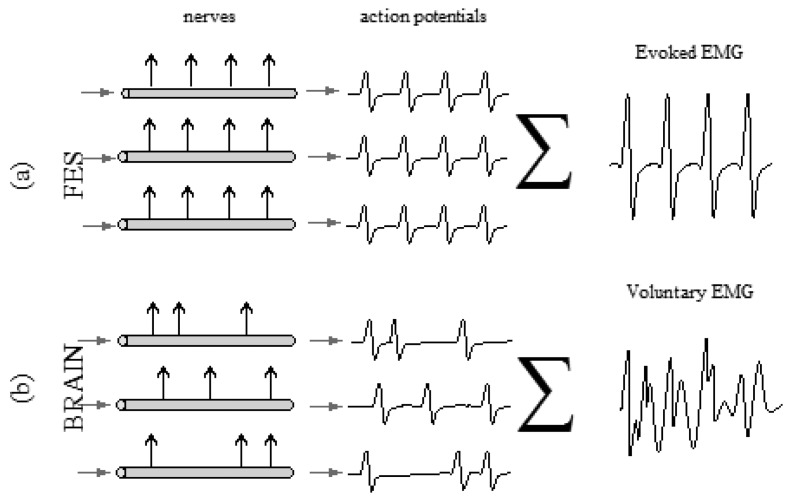
Differences between the evoked electromyographic signal (**a**) and the voluntary electromyographic signal (**b**). In the first case scenario (a), the fibres are activated by FES in a synchronous manner, building up a repetitive curve, also known as an M-wave.

In terms of signal characteristics, the variables of electromyographic signals from electrically evoked contractions exhibit smaller fluctuations when compared to that of voluntary contractions [15]. The eEMG signal is a repetitive waveform resulting from the synchronous firing of all motor units in response to the FES stimulus [77], unlike in a voluntary contraction (Figure 4). Another difference between the signal conditioning of electrically evoked and voluntary EMG signals is the critical need to remove the stimulation artifact in the former during analysis [78]. Efforts to limit the effect of the stimulation artifact include the usage of blanking window to extract M-wave form the raw eEMG [30]. In terms of the muscle force estimation, when compared to that of a voluntary contraction, the variances of the force output in an electrically evoked muscle contraction were found to be similar. Therefore, motivational changes are less important than physiological changes [79]. This supports the importance of the force output quantification during the application of an electrically evoked or voluntary electromyogram to monitor muscle function and performance [78].

### 3.6. Relationship between an Evoked Muscle Contraction and M-Wave

**Figure 5.**
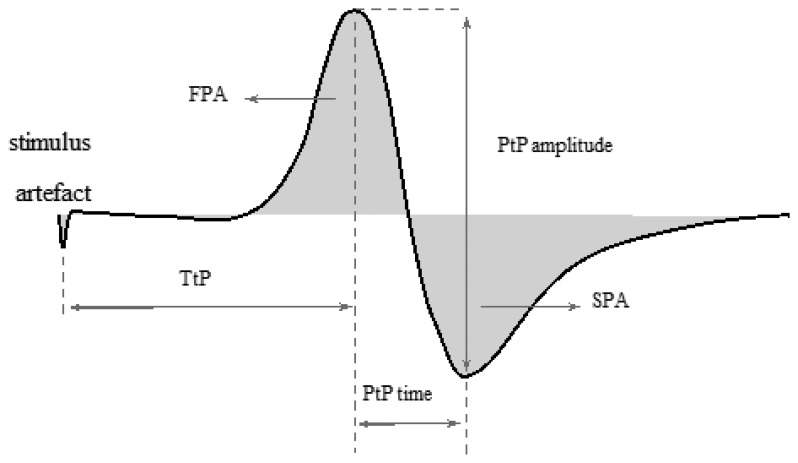
The general representation of M-wave and some of the commonly extracted parameters: peak-to-peak amplitude (PtP), time between peaks (PtP time), time to peak (TtP), first peak area (FPA), second peak area (SPA) [51]. Typical, in time domain, the amplitude variables of an M-wave are voltage values detected from specific points of the signal, normally the peaks. The M-wave amplitude is essentially a reflex of the magnitude of the sum of individual Motor Unit Action Potentials (MUAPs) [83].

The fact that M-wave parameters vary as the contraction progresses led to the investigation on the relationship between stimuli evoked contraction and EMG evoked parameters. Bigland-Ritchie *et al.* (1982) described the stimulus-evoked neuromuscular potentials, *i.e.*, an M-wave as a muscle fiber action potential evoked by a single supramaximal shock [80]. Unlike voluntary contractions, if axons of motor neurons are electrically evoked, motor unit action potentials are elicited and synchronized by external stimuli to produce a compound muscle action potential (CMAP), *i.e.*, an M-wave in relation to each stimulus [78]. In effect, the M-wave is the summation of all synchronous firing rates of the motor units (MU) when an EMG is detected during an electrically elicited contraction [81] (Figure 5).

M-wave detection depends on many factors including the dispersion of the innervation zone, numbers of active MUs, the conduction velocity of the MU distribution, the site of MUs within the muscle, the thickness of the subcutaneous tissue layers, the orientation of the detection electrode with respect to the muscle fiber and the shape of the intracellular action potential [82]. The M-wave has been adopted to quantitatively investigate the relationship between FES and muscle fatigue. The amplitude of MUAPs tend to decrease with the muscle fatigue because some of the fatigued motor units have been reported to cease firing during fatigue [83]. Hence, the respective innervation fibers cease to contract and do not contribute to the total muscle force. Consequently, the reduction in the M-wave amplitude will be manifested as a reduction in the contributing MUAPs [84]. Estigoni *et al.* (2011) reported a consistent correlation between the muscle torque-time curve and the M-wave while investigating the relationship between FES-evoked cycling and the associated changes in an evoked EMG signal [51]. Kiryu and colleagues equally validated the relevance of the M-wave to study the muscle fatigue. The investigators verified that the mean power frequency (MPF) and the instantaneous frequency were correlated to fatigue. However, as fatigue progressed, the correlation was negative [52]. Collectively, the variables of the M-wave including the temporal PTP amplitude, mean absolute value (MAV) amplitude, peak amplitude (PA), RMS, rise time to peak (RTP) amplitude and spectral features including the median frequency (MDF) and MPF, have been specifically used to monitor FES induced muscle activities in order to investigate the propagation pattern of MUAP in muscle fibers during contraction [3] (Tables 1 and 2).

**Figure 6.**
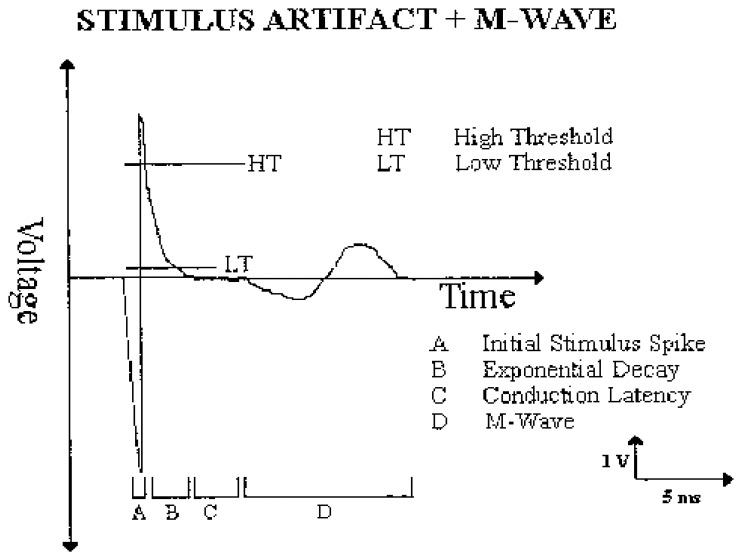
An example of the effect of stimulation artefact on evoked EMG (M-wave) [88].

However, the reliability of the relationship remains questionable especially in a transcutaneous stimulation with a surface detection electrode because of the issue of stimulation artifact [78] (Figure 6). The stimulation artifact often overwhelms the evoked EMG signal during recording [53] and saturates the amplifier during surface stimulation [85]. Erez and colleagues determined a generalized solution to the stimulus artifact removal, but the effectiveness of their framework on M-wave parameters was not validated [86]. Yi and colleagues investigated the efficacy of the stimulus artifact blanking during an EMG-triggered stimulation. The investigators highlighted issues of accuracy, the stimulation protocol, the self-adaptability and the circuit portability as areas requiring additional research [87]. In general, while stimulus-evoked fatigue may be tracked by the parameters of an M-wave derived from an eEMG, investigators had to continuously contend with the critical issue of a stimulation artifact [3].

### 3.7. Clinical Implications of an Evoked EMG as a Biofeedback Descriptor of an FES-Evoked Contraction

The efficacy of a closed loop controlled FES has been acknowledged, but its use in clinical practice has not been widely accepted [89]. This explains why the less efficient open loop FES modality is still the accepted clinical practice. The clinical relevance of every successful study on FES-evoked activity is an objective that is more important than experimental validation. Evoked EMGs have been verified to indicate muscle conditions and to track fatigue, thereby serving as a biopotential and potential feedback signal in the automatic modulation of the FES parameter as evident in the availability of several models [90]. The intervention of a biopotential-controlled FES application for individuals with a neuromuscular disability has been suggested to ensure a consistent and adequate muscle force in order to: improve neuromuscular control, increase muscle functionality and enhance motor relearning [91]. Apart from the well-known approach to mitigate the effect of fatigue using the alteration of stimulation parameters [74,92], if an effective modulation of FES control parameters is achieved through the underlying biopotentials (eEMG), muscle fatigue could equally be controlled. The feedback information of the muscle force development through a biopotential via an intact peripheral nervous system is required to modulate muscle activities and to produce an optimal and smooth electrically-induced muscle activation [46].

The use of an eEMG as biofeedback in a controlled FES application in SCI individuals has been demonstrated to replace the lost proprioception [93]. Such models have been demonstrated to show clinical effectiveness [94]. Currently, only an approximate level of muscle activity can be assessed with this modality. The accurate quantification of the entire set of physiological parameters [95], due to the complexity involved in the analysis and interpretation of an M-wave during muscle activities, remains elusive [83]. The studies on the efficacy of evoked EMGs to assess muscle activities were often reported with minimal considerations to its clinical relevance. Few experimental trials were reported on dynamic muscle actions, which is central to an assessment of muscle force and fatigue during functional human activities [96].

It is vital to emphasize that this effort is not meant to circumvent the significant ability of eEMG modality to assess peripheral properties of the neuromuscular system, but rather to highlight the limitations of its significance, especially in patient management. In terms of its clinical efficacy, the available evidence is insufficient to suggest that eEMGs can sufficiently quantify the force or predict fatigue to optimize the effectiveness of FES in individuals with SCI.

## 4. Discussion

The fact that only sixteen retrieved citations specifically investigated the efficacy of muscle force quantification and fatigue assessment with evoked EMG parameters in SCI populations suggests inadequate experimental and clinical trials. It can be inferred from the current state of evidence that the conventional FES is yet to reach its potential until less fatiguing activities and a precise control of generated force are achieved. Such a system may be realized with a biofeedback-controlled FES pattern where the state of the limbs can be monitored artificially [97].

It is evident that an evoked EMG signal may not sufficiently assess muscle force and fatigue during dynamic activities. However, a reliable estimation has been demonstrated during an isometric contraction using amplitude-based parameters with significant care during the signal acquisition stage. Similar observations were also highlighted by Clark *et al.* (2007), on the low reliability of an EMG to assess human neuromuscular functions [98], Braz *et al.* (2009), on the insufficient ability of an eEMG to predict muscle fatigue [53] and by Jiang and colleagues, on the limited clinical and commercial impact of an eEMG to control an artificial limb [99].

Evoked EMG may be characterized by a less stimulating artifact when an implanted stimulation is used [10]. Therefore, the first step in the analysis of the eEMG is the removal of artifact. Consequently, during the acquisition of a surface M-wave during transcutaneous FES stimulation, the effect of the stimulation artifact is practically unavoidable because it is caused by the potential difference between the eEMG detection electrodes and the stimulating current [77]. Thus, the signal may assess the force and monitor fatigue only with a dedicated electrical circuit to reduce the stimulation artifact (Tables 1 and 2).

A generalization of the observed trend cannot be made at this stage due to insufficient evidence and a lack of randomized control trials. Many of the reviewed trials were based on experiments with small sample sizes and an even smaller number of paraplegia subjects (Tables 1 and 2), which may reduce the external validity of the research findings. The exclusion of non-human subjects and the inclusion of studies reported in English language only may have limited the ability for a generalization. Thus, this study highlighted the lack of sufficient evidence to uphold the efficacy of muscle force and fatigue prediction during FES-evoked activities using evoked EMG parameters. The need for multi-centered randomized controlled intervention studies to ascertain the effectiveness of the modality is warranted to guide clinical management decisions.

## 5. Conclusions

Collectively, the M-wave variables were reported to change with the progression of muscle fatigue, confirming h1 to be true. Upon closer observation, h2 could be suggested to be inconsistent under dynamic conditions but is likely to be true for M-wave variables under isometric contractions. We found h3 to be false based on the results extracted from the articles that highlighted the differences in the recruitment strategy of the paralyzed and healthy muscles. This implies that healthy subject studies cannot be used to make decisions on SCI FES fatigue protocols and assessment. Because the muscle fatigue mechanism is not entirely assessed by using the eEMG potentials, the M-waves could only serve as an indicator of muscle fatigue under strict conditions. Thus, h4 is validated under such strict experimental conditions. The trend of the studies to use an evoked EMG to assess FES-evoked muscle performance generally showed that investigators had to trade-off (i) aesthetics or a compact design for a complex electrical circuit to remove the stimulation artifact and (ii) transcutaneous electrodes for invasive percutaneous or implanted stimulation electrodes for useful parameters of an M-wave (eEMG) to be derived. The robustness, optimization and safety of the future FES activities appear to be dependent on its sensitivity to the evoked muscle force and reduction of fatigue occurrence. Although the current level of prediction accuracy of the muscle force and fatigue using M-wave parameters to enhance the efficacy of an FES application for SCI individuals is insufficient, however, the state of evidence does not equally invalidate the application of the eEMG as a biofeedback descriptor. The possibilities in this field are significant; however, an extensive amount of work needs to be conducted to characterize the eEMG signal as a practical proxy of muscle force and fatigue with applications in clinical practices and general FES rehabilitation of SCI individuals.

## Figures and Tables

**Table 1.** Summary of studies on electrically evoked contraction and evoked electromyographic (EMG)-force relationship.

**Reference**	**Clinical Population and Study Design**	**Parameter Analysed**	**Outcome/Findings**
Mizrahi *et al.*, 1997 [17]	1-T4 and 1-T6/7 SCI individuals Transcutaneous isometric stimulation of quadriceps muscle with PW of 0.25 ms SF of 20 Hz 16 weeks of 45 min stimulation per week Level of Intramuscular pH was used to represent fatigue within the contractile element of the muscle model Measured at primary and post-recovery fatigue stage.	PTP, RTP, AVREC, RMS, TSP, MDF	Force-eEMG relationship was correlated by PTP and RMS (*r* = 0.97 and *r* = 0.95, *p* < 0.05) in fatigue and post fatigue period, respectively. Force-eEMG parameters showed that metabolic and electrolytic factor may be significant in assessing recovery and fatigue. IM pH decreased to 6.2 and correlated to the decay in the stimulated quadriceps force.

Erfanian *et al.*, 1998 [43]	2 complete T7 SCI individuals VL was activated under isometric condition at one joint angle Artefact balancing was used to remove stimulation artefact 6 different percutaneous stimulation patterns were adopted Constant SF and amplitude of 20 Hz and 20 mA was used, respectively.	MAV of eEMG	Evoked EMG predicted muscle torque at only one angle. Mean square error (MSE) of 0.0383 was obtained as performance index and showed the quality of prediction. The approach is only viable for intramuscular stimulation. No verification yet on multi-muscle, *i.e.*, FES practical use.

Ding *et al.*, (2005) [44]	14 SCI (all except one has thoracic level motor compete lesion). Transcutaneous stimulation was used to evoke isometric force of the quadriceps femoris muscle. Each subject participated in pre-fatigue (1 stimulation train every 20 s) and after 10 m rest fatigue protocol (110, 13-pulse, 40 Hz trains, *i.e.*, fatigue inducing train). Stimulation trains were delivered with a 700 ms rest time between successful trains.	PF, FTI	The predictive model was recommended for FES application because of the rapid parameter identification, fast optimization analysis and accurate prediction for feedforward control. The ICCs between the experimental and predicted force-time integrals and peak forces were above *r* = 0.90, *p* = 0.05. However, the model could only predict the force response of quadriceps at one length, under isometric contraction at only one knee joint angle. Prediction of muscle force for real time FES functional activities was only recommended.

Zhang *et al.*, 2011 [45]	5 SCI individuals (3-T6, 1-C5, 1-C7) Right triceps surae muscle group was activated to generate isometric ankle torque with constant SF of 30 Hz and PW of 0.45 ms Surface stimulation was adopted to plantarflex the ankle joint Measurement taken during: fatigue inducing test, fatigue recovery test and random test	MAV of eEMG	Torque prediction model based on Hammerstein structure properly fitted muscle model under isometric condition. With 18 s prediction horizon, RMS and Peak prediction errors were 0.097 and 0.34 maximum, respectively. Dynamic muscle action is necessary to validate the model for FES practical application. Reliability of the model was not investigated.

Hayashibe *et al.*, 2011 [10]	1 complete T8 SCI individual Quadriceps and tibialis anterior muscles were activated by an implanted neural stimulation of peroneal nerves with the fixed SF of 30 Hz and PW of 0.6 ms Stimulation strategy was chosen to induce high level of fatigue Dynamometer was used to measure the torque of isometric ankle dorsiflexion	MDF, MAV of M-wave	MDF of M-wave was correlated with the torque during fatigue (*r* = 0.77, p < 0.05) but not during potentiation Normalised RMS deviation was 0.145 and 0.00884 for the prediction indices. Torque prediction at low magnitude of EMG was less accurate. The study suggested significant relationship between M-wave and torque only if implanted stimulation is used.

Li *et al.*, 2012 [6]	2 T6 level SCI individual used for validation of the muscle model Transcutaneous stimulated with the SF of 30 Hz and PW of 0.45 ms was used during isometric condition The relationship between eEMG and torque/force with Non-linear Arm type recurrent neural network (NARX-RNN) model was demonstrated.	MAV of eEMG, Muscle torque	For prediction horizons of (10, 50, 70 s) the RMS error ranges from 0.0402 to 0.1067 Model performance on muscle dynamic action was not verified.

Hwang *et al.*, 2012 [46]	4 incomplete SCI and 4 Healthy Volunteers Transcutaneous stimulation of fixed SF of 20 Hz and PW of either 0.4 ms or 0.8 ms was adopted to activate quadriceps muscle group VEMG, eEMG and the combination of both were measured separately to determine the contribution of each to force/torque estimation RBF neural network algorithm adopted to assess torque using parameters derived from VEMG and eEMG	RMS of VEMG, M-wave of eEMG	RMS of VEMG was shown to estimate torque but the performance was poor during validation in the feedback control system. The processing speed was low for an on line control application.

Abbreviation: PW: Pulse width; SF: Stimulation frequency; VL: Vastus lateralis; PTP: Peak to peak amplitude; RTP: Rise time to peak amplitude; AVREC: Average rectified; RMS: Root mean square; TSP: Total spectra power; MDF: Median frequency; MAV: Mean absolute value; Peak torque: PTV; Force-time integral: FTI; EMG: Voluntary EMG; eEMG: Evoked EMG; RBF: Radial basis function; IM: Intramuscular.

**Table 2.** Summary of studies on electrically evoked contraction and evoked EMG-fatigue relationship.

**Reference**	**Clinical Population and Study Design**	**Parameter Analysed**	**Outcome/Findings**
Mizrahi *et al.*, 1994 [3]	4 complete SCI individuals Surface stimulation was administered at the motor point to evoke isometric contraction of the quadriceps muscle for maximal stimulus with the SF of 20 Hz and PW of 0.25 ms Hip and knee angle were held constant at 90° and 30°, respectively, for each participant	PTP amplitude of M-wave	The correlation coefficient of PTP amplitude of eEMG and force was up to (*r* = 0.90) during fatigue. Circuit designed to suppress stimulation artefact led to another form of noise called electrode offset potential of higher magnitude than M-wave.

Erfanian *et al.*, 1996 [54]	1 complete SCI individual Six minutes of a sustained percutaneous stimulation was used to activate vastus lateralis under isometric condition at 30° flexion and 0° extension knee angles The SF was 20 Hz and constant amplitude of 20 mA.	PA, MDF	PA and the power spectrum increased during potentiation, decreased during fatigue and increased again during maximal fatigue Postactivation potentiation, fatigue, and maximal fatigue states were manifested after a prolonged stimulation.

Tetapac *et al.*, 1997 [40]	4 complete SCI and 2 healthy individuals The subject were considered untrained for FES contraction Surface stimulation with the SF of 25 Hz, and PW of 0.25 ms was administered at the motor point of the wrist flexor for maximal stimulus under isometric muscle action. Information about FES-induced fatigue was derived from the force decline as shown on torque *versus* EMG curve.	MDF, MAV, PTP, RMS	The drop in MDF gave an indication of fatigue due to the neuromuscular propagation. Force level varied between 62% and 96% between initial (non-fatigue) and end of recovery stage. Implications on the dynamic contraction and prolonged fatigue were not described.

Chesler & Durfee, 1997 [55]	3 SCI and 20 healthy individuals 1 h per day, 5 days a week of an isometric quadriceps muscle strengthening by a transcutaneous stimulation with the SF of 30 Hz and PW of 0.3 ms was administered	RMS, MF, MAV	Noiseless eEMG was difficult to obtain, thus, limited the usage in FES practical application. Amplitude based parameters of eEMG were more relevant than the frequency based as indicator of muscle fatigue.

Chen & Yu, 1997 [49]	4 Complete SCI individuals Surface electrical stimulation of SF of 20 Hz and PW of 0.3 ms and surface eEMG acquisition were adopted Fatigue protocol was conducted on cycle ergometer. Only quadriceps muscles were stimulated in isolated stage while quadriceps and hamstring were stimulated in reciprocal fashion.	PTP amplitude	During continuous and intermittent stimulation there were positive correlation between PTP of eEMG and muscle force (*r* = 0.94) and (*r* = 0.78), respectively. The decrease of PTP of EMG from a maximum value of 0.66 mV to an asymptotic value of 0.5 mV signified the metric of fatigue. The investigators suggested that evoked EMG may not be sensitive to fatigue during dynamic contraction because of the larger inflection time and the time constant of the PTP of evoked EMG.

Yu *et al.*, 1999 [56]	5 SCI individual with lesion between C7-T11 Transcutaneous stimulation of the quadriceps was used with monophasic WF of frequency- 20 Hz, PW- 300 ms and maximum current was 120 mA eEMG was obtained from the muscle belly of the quadriceps To induce fatigue; an isometric and dynamic muscle actions were administered between 30° and 110° of knee flexion at 30°/s for dynamic contraction.	PTP amplitude, RTP, PTP duration, and torque	During fatigue; the decline in the PTP was positively correlated with the decline in the force output (*r* = 0.88, p < 0.05) while the temporal features RTP and PTP duration were negatively correlated with the decline in the torque (*r* = −0.74 and −0.73, p < 0.05) respectively. The decaying rate of the temporal feature and the torque output were slower in isometric contraction that in dynamic, *i.e.*, dynamic contraction is more susceptible to muscle fatigue.

Heasman *et al.*, 2000 [1]	2 SCI individuals Implanted stimulator was used to activate EDC, FPL, EPL muscles with peripheral nerve stimulation of PW modulation (0–0.2 ms), constant current of 20 mA and SF of 12 Hz. Load cell was used to measure the isometric muscle force Protocol was conducted once per day, and repeated once every four weeks on a separate occasion for each participant	(PTP, RMS, SPA) amplitude, MNF	SPA and RMS of M-wave demonstrated the highest correlation (*r* = 0.88) to force during non-fatigue or fatigue state. M-wave parameters indicated muscle electrical activation, but were relatively invariant to muscle fatigue. Non-isometric contraction was not investigated.

Estigoni *et al.*, 2011 [51]	8 SCI individuals FES-cycling of 15 min duration with 5 min recovery time performed by each participant 2–3 times per week for at least 6 weeks of FES-cycling sessions before the test Transcutaneous SF of 25 Hz and PW of 0.3 ms was administered to activate quadriceps muscle eEMG was carefully acquired from rectus femoris muscle	PTP amplitude	Variation in magnitude of M-wave changes compared to torque changes disallowed statistical modelling understanding of the fatigue effect generated by M-wave curve. eEMG could not predict the decrement of muscle torque during fatiguing FES-cycling.

Li *et al.*, EMG [30]	5 SCI individuals (3-T6, 1-C5, 1-C7) Surface stimulation of the right triceps surae was delivered to plantarflex the ankle joint with the constant frequency (30 Hz) and constant PW (450 μs). Isometric angle planter-flexion torque was acquired. Each subject performed both fatigue-inducing (1 s ramp up, 2 s plateau, 1 s ramp down then 2 s rest) and random tests. Maximum stimulation amplitude was set at the point where the torque became saturated for each subject.	MAV, Torque	The NARX-RNN demonstrated a robust identification performance while keeping its accuracy and stability. Future work to verify the performance of the model on the adaptive closed loop FES control for dynamic motion based on eEMG and angle-velocity sensing. Due to the variability in the subjects' level of neurological lesion, subject-specific model may be more suitable.

Abbreviations: PW: Pulse width; SF: Stimulation frequency; PTP: Peak to peak amplitude; PA: Peak amplitude; RMS: Root mean square; MDF: Median frequency; MAV: Mean absolute value; SPA: Second phase area (area under the curve of second phase of average M- wave); MNF: Mean frequency; WF: wave form; PW: Pulse duration; EDC: Extensor Digitorum Communis; EPL: Extensor Pollicis Longus; FPL- Flexor Pollicis Longus; NARX-Nonlinear autoregressive exogenous model; RNN-Recursive neural network.
